# Genetics of Plant Metabolism

**DOI:** 10.3390/ijms24086890

**Published:** 2023-04-07

**Authors:** Nicolò G. M. Cultrera

**Affiliations:** CNR-IBBR Institute of Biosciences and Bioresources, National Research Council, 70126 Bari, Italy; niccolo.cultrera@ibbr.cnr.it

This Special Issue is aimed to collect scientific papers that support holistic methodological approaches, both top-down and horizontal, for the correct application of various omics sciences because, when well-integrated, they can contribute to our understanding of the genotypic plasticity of plant species.

The goal of molecular biology scientific research on agricultural crops is to use the developed technologies in order to recognize and manipulate the levels of metabolites for food applications such as nutraceutical, clinical therapeutic and pharmaceutical purposes and for the subsequent formulation of appropriate dosages, with positive effects on metabolic syndromes, including reduced risks of morbidity and mortality. In the field of bioinformatics and computational biology, high-throughput omics technologies, which can be used with tailored and even predictive approaches, such as genomics, transcriptomics, proteomics, lipidomics, metabolomics, interactomics and systems biology, are undertaking to develop and characterize specific molecular markers linked to loci of interest in order to decrease the time duration of specific breeding programs through the fast screening of thousands of genotypes for traits of interest, enabling researchers to produce intermediate and final metabolites in plants that are useful in translational biology [1,2,3].

In fact, the most readily transposable approach concerns the study of the aggregation of successive reactions in a model multienzymatic pathway within organelles (e.g., photosynthesis, fatty acids, vitamin E, etc.), because it can be implemented without drawbacks. In fact, the instability of biological systems and low efficiency of non-compartmentalized metabolic pathways are often observed both when catalyzed by spatially separated enzymes and when crowding, the formation of nanodomains and altered local viscosities have effects on biochemical processes involving proteins [4,5,6,7]. The key elements are: (a) the recognition of the transit peptide; (b) the simple reconstruction of protein folding with predictive models (Figure 1) based on crystallography and of conformational variability based on NMR studies; (c) the analysis of the generation and transmission of specific redox signals within and between cells; (d) the correctness of the mature protein chain and possible modifications of the amino acids; and (e) the running of the pathway in an orderly manner due to the absence of competing reactions, which lead to the subtraction of intermediates with the prodromal efficiency of the substrates which are not dispersed in the cellular environment.

Phylogenetically, organellar enzymes show significant sequence similarity (homology) between plants and cyanobacteria and/or closely related lifeforms, which facilitates the isolation of species-specific genes. Therefore, the starting point is bioinformatics for a complete in silico study of both the known gene loci (which transcribe enzymes) and the metabolites (such as substrates or end products) involved in the highlighted pathway. In particular, comparative genomics studies of orthologous genes in pathways of interest, both between and within species, that are identifiable in public databases and already functionally characterized (Figure 2), should be promoted. This analysis is also a powerful tool for defining the correct identification and nomenclature of the gene locus, structure, cDNA/cds and protein sequences, together with gene families and functional domains, gene biological functions within metabolic pathways and their catalytic activities, the terms of Gene Ontology of cellular components, gene molecular functions, and the biological processes in which genes are involved, in addition to subcellular localization [8,9].

Today, the application of genomic, biochemical and physiological approaches for enhancing our knowledge of specific metabolic pathways and their related genes, include the systemized NGS, Illumina, Ion Torrent, PacBio and nanopore technology sequencing platforms; high-throughput genome annotation for genotyping purposes; phenomics platforms; high-resolution transcriptomics; and the bioinformatic analysis of DNA sequencing data (genomic, BAC clones, amplicons), as well as RNA, micro-RNA and micro-transcriptomics via laser capture microdissection (LCM), chromatin immunoprecipitation (ChIP), in situ hybridization, proteomics, lipidomics and epigenomics. Several research consortia have pursued the aims of sequencing plant genomes and studying their structures, characterizing transcriptomes, identifying genes, regulatory elements and metabolomes, and developing new markers to enhance the potential of a species in order to re-evaluate and protect germplasms and to breed desirable genotypes required for productive, sustainable and resilient agriculture.

It is my belief that well-designed and well-executed scientific experiments in the field of plant metabolism genetics can be used to answer questions and support hypotheses more promptly and effectively than the reductive but no less important conception and execution of in silico solutions, while supporting the validity of the predictive indication of the latter.

By querying in databases, genomic sequences (also gene families) are identified from cDNA libraries based on the sequence homology of the exons. On the other hand, the certainty of identifying the two possible alleles at the gene locus of interest (tissue- and moment-specific), in both homozygosity and heterozygosity, is based on the sequence homology of the introns (Figure 1).

The cloning and sequencing of the gene loci for germplasm genotyping lead to the identification of the variable allelic composition structure and reveal the alleles carrying high or low expressions of the traits. These processes also lead to the development of SNPs (Single-Nucleotide Polymorphisms), insertion/deletion and SSR databases which can represent the precious molecular markers within a plant species for the functional genotyping of varieties which show differential gene expressions for the production of metabolites of interest, which can be defined as real QTLs [10,11,12]. It should be highlighted that in the case of many fruit tree species, it is not possible to develop isogenic lineages, as is the case for herbaceous and model plants with annual cycles and with slightly heterozygous genomes.

qRT-PCR is used to analyze different correctly collected plant and fruit tissues in different stages of development (roots, stem, leaves, flowers, exocarp, mesocarp and endocarp), allowing us to determine the relative expression levels in the plant and the loci that behave as endogenous genes (housekeeping) that could act as the best controls (Figure 3a–c). This molecular approach also allows one to evaluate and report any factors that influence the metabolic pathway on the transcriptional and post-transcriptional levels. Not only sequence data related to the gene promoter but also data on the untranscribed regions (UTRs) of the genes allow one to identify the regulatory and promoter elements that affect the physiology and, therefore, the production of metabolites of interest, as well as the defense mechanisms against endogenous and exogenous stress in the case of evolutionary adaptation (light, temperature, biological clock, micro-RNA and other related binding factors, such as the response to ethylene and the various GATA, MAD box, etc.) and the genes involved in the maintenance of genomic integrity, transcription and protein stability. These data also allow one to study the epigenetic factors of functional control of genes [8,13]. When the loci of interest of the metabolic pathway under study and the tissue under examination have been identified, specific antibodies (or probes) can be constructed for each gene locus (Figure 3d–g) in order to evaluate the relationship between the transcription of each gene and the amount of protein that is translated and produced [14].

This research combines EST-cDNA (Expressed Sequence Tag) libraries and subtractive libraries, transcriptomes and RNA-Seq data on organs/tissue that are available with the quantification of the gene expression levels (RPKM) [15,16,17]. These libraries are often not complete (in terms of coverage) or completely reliable in terms of indicating the correct contigs of the genes under study.

The data available on the gene sequences and the genetic factors that control the agronomic behavior of plants are still scarce and fragmentary, partly because the comparative genomes have not yet been obtained as secure assembling of chromosomes, especially in highly heterozygous plants with highly repeated genomes [18]. However, genomic scaffolds obtained with high-coverage DNA sequencing are available and are an important resource for functional genomics studies.

For the sake of methodological thoroughness, functional genomics studies are an attractive means of making significant progress in translational research [19]. Thus, achievements related to biotechnologies such as gene silencing and phenotype disruption analysis could also be promoted. The complete characterization of candidate genes can also easily be implemented in biotechnological experiments examining the overexpression of genomic cDNA in transgenic plants through transformations mediated by agrobacteria. Today, different innovative biotechnologies are available for the study of heterologous gene expression in model plants, yeasts and bacteria, including the modification of genes and/or their insertion into nuclear or plastid DNA, whether through the transformation of plastomes using biolistic approaches [20], by reconstructing the biosynthetic pathways in engineered yeast and/or by gene transfer through precision “breeding” interventions with biotechnologies such as cisgenesis, CRISPR/Cas9 technology, etc.

The applications of multiple molecular biology, computational and bioinformatics technologies in the scientific contributions accepted for publication in this Special Issue offer peculiar/specific demonstrations of the implementation of various desired and correct macro-approaches.

## Figures and Tables

**Figure 1:**
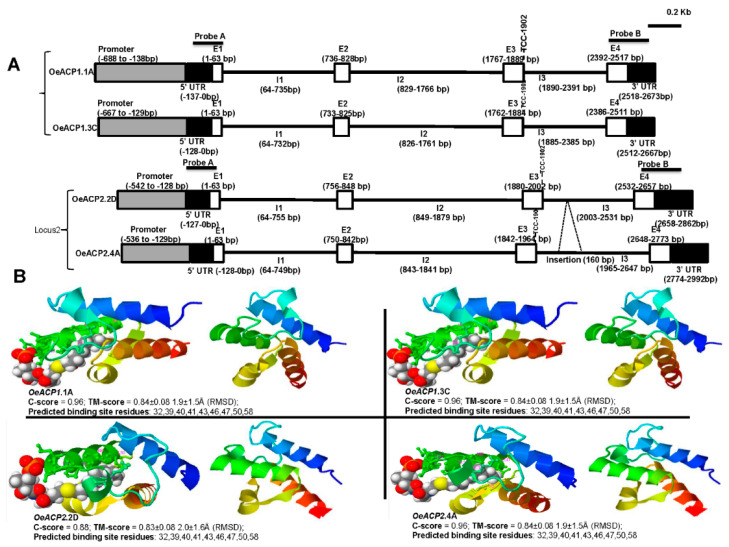
Structure of ACP loci. (**A**) Genetic structure. (**B**) Protein folding, structural models and binding site residues predicted by I-TASSER. C-score for estimating the quality; TM-score and RMSD as standards for the accuracy of structural modeling.

**Figure 2:**
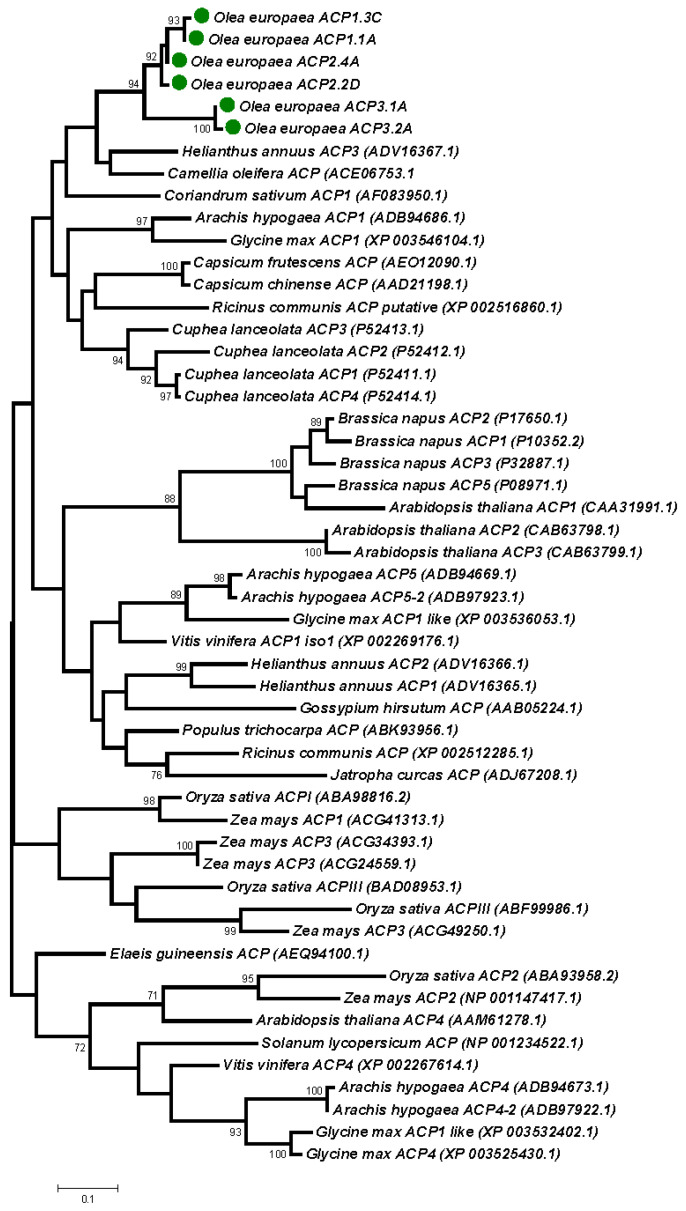
Comparative Neighbor Joining phylogenetic tree of orthologous genes for the pathway of interest.

**Figure 3:**
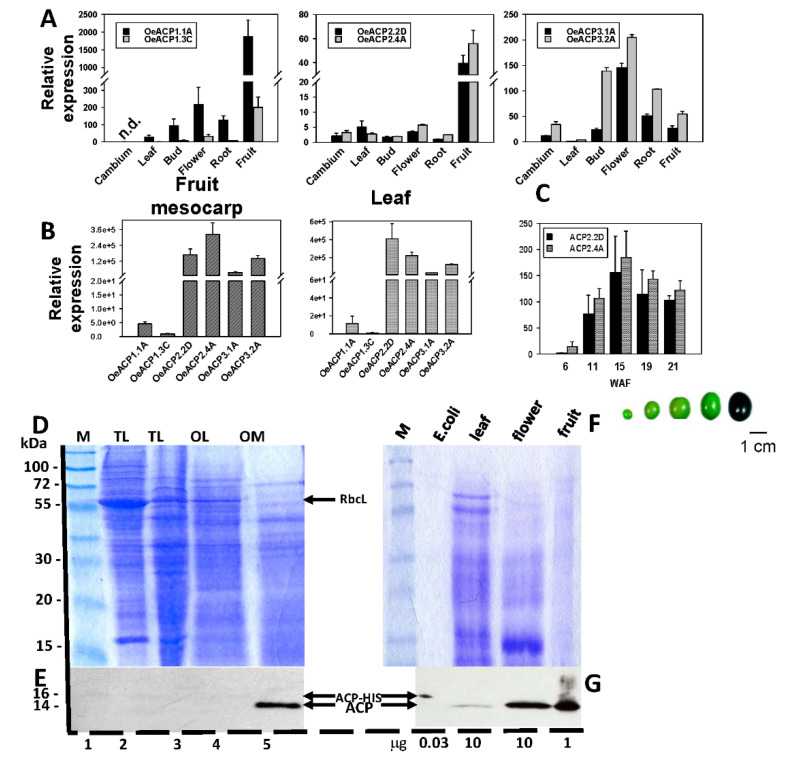
(**A**) Relative abundance of OeACP transcripts in different olive tissues, indicating significant differences between alleles belonging to the same locus. (**B**) Relative abundance among all the ACP transcripts (compared in mesocarp and leaf). (**C**) Relative transcript abundance of locus OeACP2, alleles 2D and 4A in the mesocarp during fruit development, with statistical significance. (**D**) Separation of olive proteins by SDS-PAGE and specificity of the antiACP antibody: proteins extracted from tobacco leaf (TL), olive leaf (OL) and olive mesocarp (OM), with molecular mass markers (M). (**E**) Western blot analysis using anti-ACP antiserum on 10 μg of leaf protein from several plant species. Separation by SDS-PAGE in two identical gels: (**F**) abundance of the ACP protein in different olive tissues with Western blot analysis using anti-ACP antiserum (the amount of proteins loaded in each lane, in µg, is indicated under the figure); (**G**) stained gel with 0.1% Coomassie brilliant blue. Affinity-purified His-tagged ACP from *Escherichia coli* was used as a positive control. Numbers on the left indicate the positions of molecular mass markers in kilodaltons.
